# Supplementary material for: Coupling of Slack and NaV1.6 sensitizes Slack to quinidine blockade and guides anti-seizure strategy development
Source: eLife. 2024 Jan 30;12:RP87559. doi: 10.7554/eLife.87559 (PMC10942592; doi:10.7554/eLife.87559)
Supplement: Supplementary file 1. — (a) The sensitivity of Slack to quinidine blockade upon expression of Slack alone and co-expression of Slack with NaV1.x. (b) The sensitivity of NaV channel subtypes to quinidine blockade upon expression of NaV1.x alone and co-expression of NaV1.x with Slack. (c) Biophysical characteristics of NaV1.6 expressed alone and NaV1.6 upon co-expression with Slack. (d) The sensitivity of Slack mutant variants to quinidine blockade upon expression of Slack mutant variants alone and co-expression of Slack mutant variants with NaV1.6. [file elife-87559-supp1.docx]

Supplementary File 1 for

**Coupling of Slack and Na_V_1.6 sensitizes Slack to quinidine blockade and guides anti-seizure strategy development**

Tian Yuan *et al.*

*Corresponding author. Email: huangz@hsc.pku.edu.cn

**This file includes:**

Supplementary file 1a to 1d

**Supplementary file 1a. The sensitivity of Slack to quinidine blockade upon expression of Slack alone and co-expression of Slack with Na_V_1.x**

| Channels | IC_50_ (μM) | 95% CI | n |
| --- | --- | --- | --- |
| Slack | 85.13 | 58.29 to 126.8 | 6 |
| Slack(Na_V_1.1) | 24.83 | 16.36 to 26.55 | 7 |
| Slack(Na_V_1.2) | 14.83 | 7.90 to 28.30 | 10 |
| Slack(Na_V_1.3) | 23.64 | 10.88 to 50.80 | 13 |
| Slack(Na_V_1.5) | 29.46 | 11.58 to 78.67 | 9 |
| Slack(Na_V_1.6) | 0.87 | 0.58 to 1.30 | 19 |

**Supplementary file 1b. The sensitivity of Na_V_ channel subtypes to quinidine blockade upon expression of Na_V_1.x alone and co-expression of Na_V_1.x with Slack**

| Channels | IC_50_ (μM) | 95% CI | n |
| --- | --- | --- | --- |
| Na_V_1.1 | 129.84 | 115.06 to 146.52 | 5 |
| Na_V_1.2 | 79.71 | 57.75 to 110.02 | 3 |
| Na_V_1.3 | 63.77 | 52.43 to 77.56 | 6 |
| Na_V_1.5 | 35.61 | 28.88 to 43.91 | 6 |
| Na_V_1.6 | 51.50 | 35.77 to 74.15 | 4 |
| Na_V_1.1(Slack) | 105.72 | 81.10 to 137.81 | 6 |
| Na_V_1.2(Slack) | 62.04 | 49.29 to 78.11 | 3 |
| Na_V_1.3(Slack) | 94.04 | 63.64 to 138.98 | 12 |
| NaV1.5(Slack) | 40.23 | 24.18 to 66.94 | 9 |
| Na_V_1.6(Slack) | 39.41 | 28.10 to 55.28 | 5 |

**Supplementary file 1c. Biophysical characteristics of Na_V_1.6 expressed alone and Na_V_1.6 upon co-expression with Slack**

| Channels | Pipette solution | Steady-state activation | | | Steady-state fast inactivation | | |
| --- | --- | --- | --- | --- | --- | --- | --- |
|  |  | V_1/2_ (mV)  with 95%CI | k  with 95%CI | n | V_1/2_ (mV)  with 95%CI | k  with 95%CI | n |
| Na_V_1.6 | K-gluconate-based | -7.38  (-8.36 to -6.40) | 6.69  (5.83 to 7.56) | 10 | -48.71  (-49.37 to -48.06) | 6.04  (5.46 to 6.62) | 11 |
| Na_V_1.6 (Slack) | K-gluconate-based | -8.52  (-9.63 to -7.41) | 7.06  (6.08 to 8.04) | 6 | -49.48  (-50.34 to -48.62) | 6.07  (5.30 to 6.83) | 7 |
| Na_V_1.6 | CsF-based | -27.03  (-29.26 to -24.79) | 7.06  (5.09 to 9.03) | 5 | -63.35  (-64.86 to -61.83) | 6.52  (5.19 to 7.85) | 5 |

**Supplementary file 1d. The sensitivity of Slack mutant variants to quinidine blockade upon expression of Slack mutant variants alone and co-expression of Slack mutant variants with Na_V_1.6**

| Channels | IC_50_ (μM) | 95%CI | n |
| --- | --- | --- | --- |
| Slack^K629N^ | 19.01 | 12.50 to 28.90 | 8 |
| Slack^K629N^(Na_V_1.6) | 0.26 | 0.09 to 0.73 | 8 |
| Slack^R950Q^ | 25.06 | 13.12 to 47.89 | 7 |
| Slack^R950Q^(Na_V_1.6) | 0.34 | 0.13 to 0.85 | 5 |
| Slack^K985N^ | 46.10 | 31.52 to 67.42 | 5 |
| Slack^K985N^(Na_V_1.6) | 2.41 | 1.52 to 3.80 | 7 |
